# Causal effects of sedentary breaks on affective and cognitive parameters in daily life: a within-person encouragement design

**DOI:** 10.1038/s44184-024-00113-7

**Published:** 2024-12-21

**Authors:** Marco Giurgiu, Irina Timm, Ulrich W. Ebner-Priemer, Florian Schmiedek, Andreas B. Neubauer

**Affiliations:** 1 Institute of Sports and Sports Science, Karlsruhe Institute of Technology (KIT), Karlsruhe, Germany; 2 Department of Psychiatry and Psychotherapy, Central Institute of Mental Health, University of Heidelberg, Medical Faculty Mannheim, Mannheim, Germany; 3 DIPF | Leibniz Institute for Research and Information in Education, Frankfurt am Main, Germany; 4 Institute of Psychology, RWTH Aachen University, Aachen, Germany

**Keywords:** Human behaviour, Risk factors

## Abstract

Understanding the complex relationship between sedentary breaks, affective well-being and cognition in daily life is critical as modern lifestyles are increasingly characterized by sedentary behavior. Consequently, the World Health Organization, with its slogan “every move counts”, emphasizes a central public health goal: reducing daily time spent in sedentary behavior. Previous studies have provided evidence that short sedentary breaks are feasible to integrate into daily life and can improve affective and cognitive parameters. However, observational studies do not allow for causal interpretation. To overcome this limitation, we conducted the first empirical study that integrated the within-person encouragement approach to test the causal effects of short 3-min sedentary breaks on affective and cognitive parameters in daily life. The results suggest that brief sedentary breaks may have a beneficial impact on valence and energetic arousal. Moreover, our methodological approach powerfully demonstrated the possibility of moving towards causal effects in everyday life.

## Introduction

Modern human lifestyles are predominantly characterized by low levels of physical activity. Due to social and environmental changes, resulting from the replacement of physical activity by technical aids in almost all areas of life, including work, transportation, and household tasks^1^, humans have evolved a lifestyle with the tendency to avoid unnecessary physical activities^2^. The precarious nature of this situation is that being physically inactive is the fourth leading risk factor for human mortality^3^. Approximately 28% of adults^4^ and 81% of adolescents^5^ worldwide do not meet current physical activity guidelines^6^. Additionally, scientific evidence over the last decade has shown that sedentary behavior (i.e., sitting/lying with minimal energy expenditure^7^) has severe negative impacts on both physiological and mental health^8–10^. Therefore, the World Health Organization recommends limiting daily sedentary time and avoiding uninterrupted sedentary bouts. In essence, it is vital to break up sedentary behavior and be active whenever possible^6^.

Due to the significant impact of physical inactivity, researchers have long studied its determinants, primarily through two frameworks: social-cognitive models (e.g., theory of planned behavior) and humanistic models (e.g., self-determination theory)^11^. While these frameworks have been essential in explaining why some individuals are active and others are not, they account for only a modest portion of the variance in physical activity and sedentary behavior^12,13^. To fully understand the impact of sedentary behavior and its interruptions on human functioning, it is essential to consider both cognitive and affective factors. This approach aligns with dual-process models, which suggest that behavior and decision-making are influenced by both an automatic, affect-based system and a deliberate, cognitively-controlled system^14,15^.

Understanding the mechanisms behind affective responses to physical activity may offer valuable insights into positive reinforcement, which can strongly predict both current and future participation in physical activity^16^. Historically, research on affective responses to physical activity has been predominantly conducted in laboratory settings^17–19^. For instance, Ekkekakis et al.^17^ provided valuable insights into how different intensities and durations of exercise influence acute affective states. In the context of sedentary breaks, Nagy et al.^20^ explored the affective responses of healthy-weight and overweight/obese children after screen-time breaks, finding that both groups experienced improved affective states immediately following the break. However, these findings may not be fully transferable to real-life contexts as they are limited in their ecological validity. Over the last decade, a growing number of studies focused on the momentary association between physical activity, sedentary behavior, and affective well-being in daily life^21,22^. The majority of these ambulatory assessment studies employ fixed or random sampling designs and analyzed aggregated patterns (e.g., between 5 min and 10 h) before the assessment of affective states; thus, providing limited informative value of the immediate affective responses to physical activity. Here, the artificial setting allows laboratory studies to accurately measure the affect response during physical activity. To approach this challenge in everyday studies is the utilization of wearable-triggered e‐diaries. Wearables monitor and analyze accelerometer data continuously in real-time and electronic diary (e-diaries) questions are triggered during sedentary^23^ or physically active phases^24^. Thus, to gain deeper insights into the complex interplay between sedentary breaks, cognitive parameters, and affective states, leveraging the methodological advancements of mobile and electronic devices presents a sophisticated approach.

Accelerometer-based measures of physical activity and sedentary behavior have become increasingly affordable and less obtrusive, resulting in their more frequent use across various research domains^25^. Continuous and passive monitoring of accelerometer data can be further combined by integrating ecological momentary assessment or experience sampling techniques such as self-reported momentary psychological states or cognitive performance tasks via e-diaries on smartphones^26,27^. This approach, known as ambulatory assessment, represents the state‐of‐the‐art methodology for examining within‐person associations in everyday life, utilizing real‐time, objective, device‐based methods and repeated measurements with a high sampling frequency.

Regular physical activity is well-established to have cognitive benefits, enhancing brain health and function across the lifespan^28^. However, the effects of more acute changes in physical activity patterns, such as interrupting sedentary behavior, are less clear. Recent research has begun to illuminate the complex relationship between sedentary behavior and cognitive performance^10^. Prolonged sitting has been associated with cognitive impairments and declines in cognitive function^29^, and, high levels of sedentary behavior have been unfavorably linked to various health outcomes, including physical, mental, and cognitive well-being^30^. In contrast, Chueh et al.^31^ reviewed seven studies involving 168 participants aged 18–80 years and found that interrupting sitting with short bouts of moderate-to-vigorous physical activity had positive effects on cognition^31^. Building on this, Feter et al.^32^ conducted a systematic review and meta-analysis of 25 randomized controlled trials (*n* = 1.289), revealing that acutely interrupting continuous sedentary time with multiple physical activity bouts improved cognitive function^32^. Similarly, Garrett et al.^33^ performed a Bayesian meta-analysis of 651 effect sizes from 113 studies with 4.390 young adult participants. They also found a small but significant beneficial effect of acute exercise on cognition^33^.

Our study focuses on the impact of breaking up prolonged sitting on executive functions, particularly working memory—the ability to hold and manipulate information mentally, such as in problem-solving^34^. The current evidence indicates that breaking up sedentary behavior by incorporating short bouts of physical activity can enhance attention, memory, and executive function, potentially by increasing cerebral blood flow, neurotransmitter release, and activation of brain regions associated with cognition^35,36^, further research is needed to clarify the ideal patterns for taking sedentary breaks during prolonged sitting to provide an immediate boost to cognitive performance.

Recent technological advancements have made it feasible to use smartphone-based cognitive tasks to measure individual variations in cognitive performance across different contexts. These mobile assessments maintain sound psychometric properties^27,37^ while capturing the dynamic nature of cognitive function in real-world settings. Ambulatory assessment studies that combined accelerometer outputs with self-reported psychological states in daily life increased tremendously over the last years^38^. For example, a recent review identified 66 studies that focused on the momentary association between physical activity, sedentary behavior, and affective states and identified as one main finding that short physically active bouts are positively related to affective states^21^. According to the literature, there is an ongoing discussion about the operationalization of affective states (i.e., two-dimensional structure^39^ vs. three-dimensional structure^40^). Based on the analyses reported by Wilhelm and Schoebi^41^, a three-dimensional approach (including valence, energetic arousal, and calmness) is highly sensitive to capturing within-person changes in affective states.

Ambulatory assessment studies represent several methodological advances such as high ecological validity, disentangling within- and between-person associations, or a clear chronological order (e.g., short physically active bouts preceding affective well-being ratings). However, those observational studies do not allow for causal interpretation, and there remains the possibility that some of the observations may reflect confounding or reverse causation^42^. In particular, temporal precedence is a necessary but not sufficient condition for a causal effect because time-lagged associations might still be spurious due to hidden time-varying third variables. For example, if feeling sick on a particular day reduces the likelihood of being physically active and also lowers affective states, the observed relation of physical activity and affective states will be confounded, regardless of the order in which the two variables are assessed. To substantiate causal relations at the within-person level, experimental study designs in daily life are therefore needed. Building on within-person experimental designs that have been used for quite some time in single case studies (e.g., the so-called ABAB designs with an intervention being switched on and off repeatedly), there recently has been an increasing interest in, and use of, so-called *micro-randomized trials*^43^. In these, certain desirable behavior is put under experimental control at the within-person level by providing participants with relevant prompts at randomly chosen occasions. That is, instead of randomizing participants into groups (i.e., treatment and control groups), in micro-randomized trials, occasions are randomized as to whether a behavior should be shown or not (i.e., treatment and control conditions).

In existing studies employing such micro-randomized designs, the behavior itself (e.g., physical activity) has often been the outcome of interest, that is, it has been investigated to which degree the prompts are able to induce the behavior, and how people differ in the strength of such induction effects. However, oftentimes these behaviors could also be considered to be treatments that are thought to have effects on certain further outcomes, like in our case physical activity potentially having an effect on affective states. In these cases, the level of adherence to the randomized prompts becomes crucial. If this adherence is perfect and the treatment behavior therefore is under full experimental control, causal effects of the behavior on the outcome can directly be inferred. Under realistic conditions, adherence often will be less than perfect, however, as participants may not be able or willing to show the intended behavior when prompted or decide to show the behavior in situations when not prompted to.

**Fig. 1:**
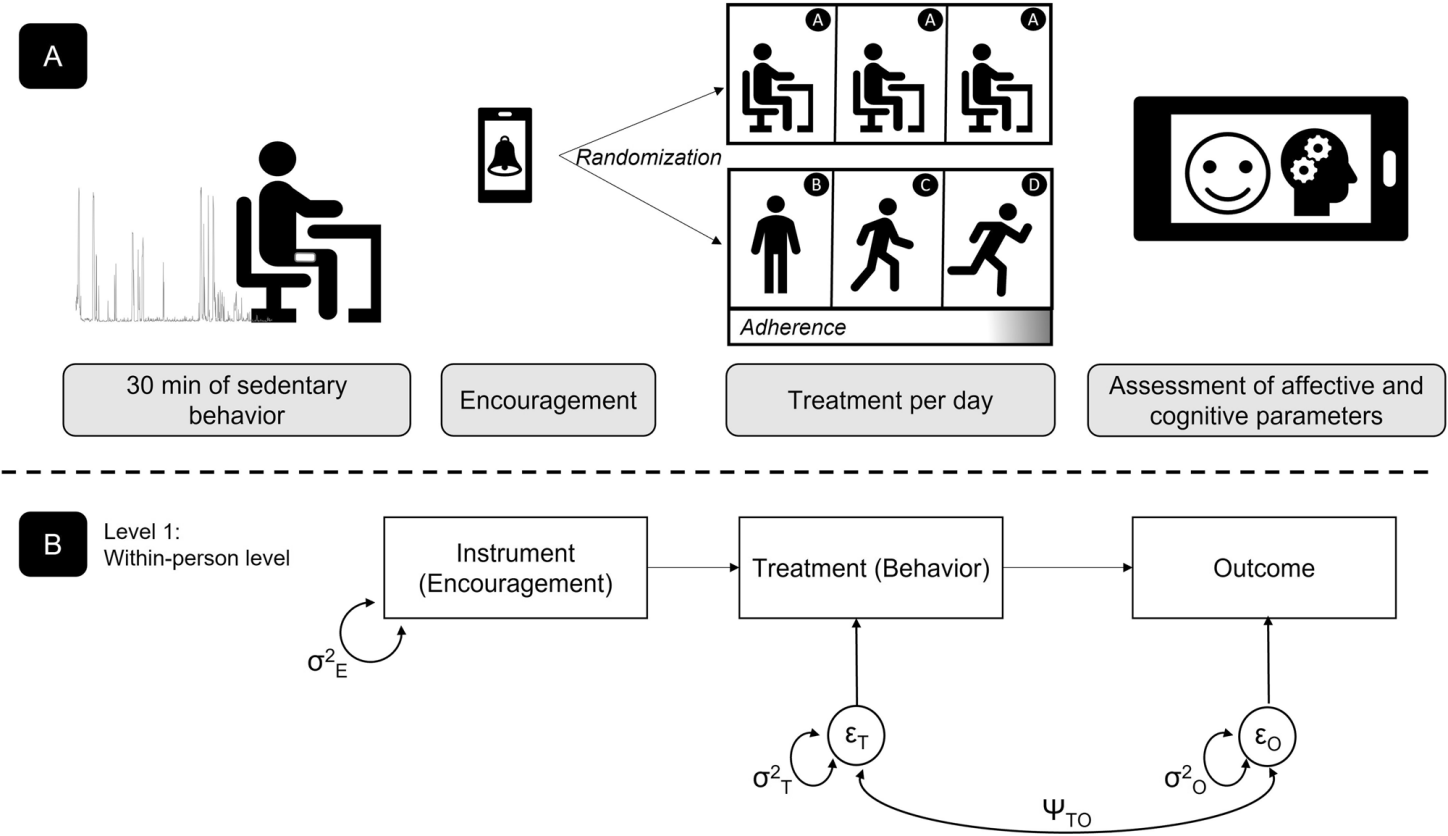
Study design. **A** The within-person encouragement design based on a wearable-triggered sedentary algorithm with randomized encouragement or non-encouragement messages, followed by either a control condition [A] or sedentary break treatment condition (standing [B], slow walking [C], fast walking [D]) with a high adherence level and subsequent assessment of affective and cognitive outcome variables. **B** The path-model on Level 1 with direct effects of the observed instrument (i.e., the encouragement condition) on the observed treatment variable (i.e., the targeted behavior) and of the treatment on the observed outcome variable is specified, together with the variances and the covariance (σ^2^_T_, σ^2^_O_, and ψ_TO_) of the residual terms of the treatment (ε_T_) and the outcome (ε_O_) at the within-person level.

In such situations, the within-person encouragement design^44^ may offer a solution. It combines the idea of micro-randomization (i.e., randomly providing or not providing encouragement to show the treatment behavior on a series of occasions) with a multilevel model implementation of instrumental variable estimation, a well-established method in econometrics for the estimation of causal effects^45^. If the two conditions are met that (a) there is at least some positive effect of the randomized encouragement on the treatment behavior (i.e., there is some adherence—the higher the better) and (b) that the encouragements (which serve as the instrumental variable) are related to the outcome only through the treatment, this method allows to estimate causal effects of the treatment behavior on the outcome—even when there are also confounding factors that influence both, treatment behavior and outcome. The basic idea here is that if there is at least some amount of random variation (induced by the randomly provided encouragements) in the treatment behavior, this variation can be used to estimate the causal effect as part of a path model (encouragement → treatment → outcome) while confounding factors are captured by allowing the residuals of treatment and outcome to correlate. Being set up as a multilevel model, this approach has the further advantage that it also allows to estimate of between-person differences (i.e., heterogeneity) in the strength of the individual causal effects and relates those differences to other variables (see the application of the design in Fig. 1).

To achieve our methodological goal, namely to provide a showcase to approach causal effects in daily life, we conducted the first empirical study that integrated the within-person encouragement approach to test the causal effects of short sedentary breaks on affective and cognitive parameters in daily life. As theoretical questions, we hypothesized, based on previous studies^21,46–48^, that compared to uninterrupted sedentary bouts (≥30 min), short 3-min sedentary breaks would have a positive causal effect on the affective state dimensions valence (Ia), energetic arousal (Ib), and a negative causal effect on calmness (Ic). Additionally, we expected a positive causal effect of the intervention on momentary working memory performance (Id). Furthermore, we expected that a higher level of activity intensity induced by the encouragement would positively predict these four outcomes (II). To gain a better understanding of between-person differences in the effectiveness of the intervention, we conducted explorative analyses on the heterogeneity of the treatment effects. Finally, we exploratively tested whether self-reported levels of well-being and sedentary behavior had changed at the beginning and after 15 days of measurement.

## Methods

### Participants

Between October 2022 and December 2023, we recruited 222 university employees, a population shown to be at high risk for sedentary behavior^49^. Potential participants who expressed interest in our study underwent a brief screening interview via email or phone to ensure they met the inclusion criteria. We included participants who spent the majority of their working day in a sedentary position (e.g., office workers or those in similar occupations), were able to perform typical daily activities without significant limitations, and did not report any current physical injuries or mental health conditions that would interfere with the study protocol. In total, 11 participants were excluded from the analyses because either they discontinued the study (*N* = 3), finalized less than 15 e-diary prompts (*N* = 7), or due to technical issues (*N* = 1). Thus, the final sample consisted of 211 participants (55% females) with a mean age of 35.6 ± 10.6 years and a mean body mass index (BMI) of 24.07 ± 3.3 kg/m^2^ (for details, see Table 1). The Ethics Committee of the Karlsruhe Institute of Technology (KIT) approved this study. All eligible participants received written and oral information regarding the study procedures before written informed consent was obtained. Participants were free to withdraw from the study at any time. Participants received a fitness tracker worth €100 as an incentive to take part.

**Table 1:** Participants’ characteristics (*N* = 211; female 55%)

Variable	Mean ± SD^a^	Minimum	Maximum
Age [yrs.]	35.61 ± 10.6	20.18	64.67
BMI [kg/m^2^]	24.07 ± 3.34	17.87	36.48
VO_2Max_ [ml/min/kg]^b^	41.33 ± 8.8	16	69
Adherence [0–100%]^c^	81.89 ± 9.8	46.3	98.72
Valence [0–100]^c^	65.59 ± 11.71	38.44	98.61
Energetic Arousal [0–100]^c^	56.55 ± 11.47	28.85	89.95
Calmness [0–100]^c^	60.55 ± 12.41	32.44	96.55
Working Memory [0–100%]^c^	88.3 ± 8.87	43.06	99.73
Wear Time Accelerometer [h/day]^d^	20.4 ± 2.7	10.63	23.74
Sitting/reclining/lying time [h/day]^d^	10.47 ± 1.3	3.86	12.88
Upright time [h/day]^d^	4.4 ± 1	2.1	7.38
Steps [number**/**day]^d^	8,533 ± 2474	3479	22,853
Movement acceleration intensity [millig/day] ^d^	77.35 ± 21.31	38.87	192.48

^a^Standard deviation.

^b^Data available from *N* = 208 participants.

^c^Aggregated per participant and day.

^d^Assessed via e-diary, aggregated per participant.

### Study procedures

After recruiting via flyers, mailing, and word of mouth, participants completed an initial in-person session of ~3 h. During this appointment, participants received written and oral information regarding the study procedures, completed a set of cognitive tasks, filled in questionnaires, and performed an incremental treadmill test to volitional exhaustion with a 6-2-1 protocol (i.e., starting with 6 km/h and changing the speed by 1 km/h after every 2 min). After the in-person session, participants started with the ambulatory assessment phase for 15 working days. If the participants were not able to participate continuously for 15 working days (e.g., due to sickness or vacation), we extended the study period until at least data from 15 working days were collected. Participants were equipped with a study smartphone (Nokia G50 or 6, Nokia Corporation, Espoo, Finland, nokia.com) and were instructed to wear the move 4 accelerometer (movisens GmbH, Karlsruhe, Germany, movisens.com) on their right thigh continuously for 24 h per day (Monday morning to Friday evening). Participants were only instructed to remove the sensor while swimming, in the sauna, or during any activity where the sensor might be hit (e.g., martial arts). The weekend days were scheduled as break days without assessments to maintain motivation and thus compliance.

We implemented the central aspects (i.e., encouragement, treatment, and outcome) of the within-person encouragement design in the movisens xs (library v7464) sampling scheme as follows. Each working day between 8:40 a.m. and 9:40 p.m., the smartphone prompted participants based on a sedentary-triggered algorithm^23^, activated after 30 consecutive minutes in a sitting/lying body position. In particular, the thigh sensor analyzed and transferred data on body position via Bluetooth Low Energy (BLE) to the smartphone in real-time. Immediately after the 30-min sedentary bout, the participants received an alert on the study smartphone with randomization to either an encouragement message (i.e., *“The next sedentary break is coming up! Take a 3-minute walking break at a relaxed pace.”*) or a non-encouragement message (i.e., “*In a few minutes, a new survey will start*.”). Over a study day, participants were prompted to complete a maximum of six momentary e-diary assessments. The treatments were *equally* balanced to 50% non-encouragement conditions (i.e., continuous being sedentary—Condition A) and 50% encouragement conditions (i.e., a 3-min sedentary break in different intensities, varying between standing [Condition B], self-selected slow walking [Condition C], or self-selected fast walking [Condition D]). Each of the six treatment conditions was randomized and selected once a day, which means that if a participant completed, for example, Condition C and answered the momentary e-diary then, in the following situations, the system randomized among the remaining five conditions (A, A, A, B, D). After the randomization and display of the treatment conditions, participants were given 5 min to complete the 3-min sedentary break or to remain in their sedentary position. One assessment point was finalized after completing the momentary e-diary ratings. Participants had the opportunity to postpone the prompts with a maximum delay of 15 min, that is three times for 5 min. Time-out phases of 30 min were integrated to minimize participant burden, which means after a completed e-diary rating, the participants were not prompted for at least 30 min. Under realistic conditions, adherence often will be less than perfect since participants may not be able or willing to show the intended behavior when prompted. Thus, during the in-person session, we asked participants to ignore prompts if they were unable or unwilling to complete e-diary questionnaire/tasks. Moreover, we were in contact with participants via smartphone messages to motivate them when adherence to the study design was low (e.g., ignoring several prompts per day). We considered adherence to a non-encouragement message met if the participant continued to spend the following 5 min in a sitting/lying position. In contrast, in the encouragement conditions (B, C, D), we considered adherence met if the participant spent at least 1 min in an upright position in the following 5 min. The adherence rate was calculated as a percentage score of being adherent. After the ambulatory assessment period concluded, participants had an in-person session where they returned the study equipment. Before as well as after the ambulatory assessment period, participants filled in questionnaires such as the World Health Organization Well-Being Index (WHO-5)^50^ or the modified version of the German Sedentary Behavior Questionnaire (mSBQ)^51^.

### Measures

#### Accelerometry

The move 4 accelerometer (movisens GmbH, Karlsruhe, Germany, movisens.com) captured movement and non-movement behaviors with a range of ±16 g and a sampling frequency of 64 Hz. Raw acceleration data were stored on an internal memory card and were processed by a band-pass filter (0.25–11 Hz) to eliminate artifacts. Previous validation studies have shown that the move accelerometer is appropriate to differentiate between sitting/lying and upright body postures^52^, and to capture intensity levels of physical activity^53^. To parameterize the accelerometer values, we calculated relevant parameters (i.e., body position and movement acceleration intensity [millig]) in 1-min intervals by using the software DataAnalyzer (version 1.16.8; movisens.com).

#### Affective state dimensions

To assess momentary affective states over time, we used a short scale consisting of six items developed and validated by Wilhelm and Schoebi^41^. The scale captures three basic affective state dimensions, including valence (V), energetic arousal (EA), and calmness (C). Psychometric properties in our sample with within-subject reliability coefficients ranging between 0.58 and 0.91. The six bipolar items were presented to participants in a reversed polarity and mixed order on visual analog scales (0–100). The items include: EA1 – tired to awake; V1 – content to discontent; C1 – agitated to calm; EA2 – full energy to without energy; V2 – unwell to well; C2 – relaxed to tense. The questions were presented on the smartphone via the application movisensXS (version 1.5.24; movisens.com).

#### Mobile working memory task

Participants completed a numerical updating task^54^. The task was set up with four digits in a horizontal array, which had to be initially memorized and then updated according to arithmetic operations (i.e., addition and subtraction in the range of ±1–8) that were presented sequentially in a random manner. The presentation time for the four starting digits was 4000 ms, followed by an inter-stimulus interval of 500 ms. In total, participants completed nine trials at each measurement occasion. During each trial, five updating operations had to be applied (i.e., each of the four digits had to be processed at least once and one digit twice) with a presentation time of 1750 ms. The presentation time for arithmetic operations was reduced by 250 ms after the 3rd and 6th trials. Participants had 4000 ms time per digit to enter the result via tapping on a digit block. At each measurement occasion, the percentage of correct responses was computed. The within-subject reliability coefficient in our sample was *ω* = 0.57. In total, the task took approximately three and a half minutes. The task was presented on the smartphone via the software Presentation (version 3.0.4; Neurobehavioral Systems, Inc., https://www.neurobs.com/).

#### E-diary intervention questions

If the participants were prompted to perform a sedentary break (Conditions B, C, D), we included a couple of self-report questions to gain insights into adherence behavior after an encouragement message. In particular, the participants were asked to answer the following four questions: i) *Did you complete the intervention?* (Response: yes or no); *ii) Where did you perform the intervention?* (Response: indoor, outdoor, or both in- and outdoor); *iii) How long did the sedentary break last?* (Response: less than 3 min, exactly 3 min, longer than 3 min); *iv) Why did you not complete the sedentary break?* (Response: meeting, no motivation, or other).

### Statistical analyses

Prior to the statistical analyses, we merged the minute-by-minute values of the accelerometer and the e-diary entries using the software DataMerger (version 1.8.0; movisens.com). To estimate within-subject reliability coefficients, we calculated McDonald’s Omega based on multilevel confirmatory factor analysis^55^. To test Hypothesis 1, we set up a multilevel model as the state-of-the-art procedure for analyzing hierarchically structured intensive longitudinal data^56^. In detail, we set up a two-level structural equation model in Mplus with repeated measurements (Level 1) nested within participants (Level 2). Intraclass correlations (ICCs) were estimated using unconditional models, including valence, energetic arousal, calmness, and working memory as outcomes. To test the proposed causal relations, we used an instrumental variable approach as an analytical framework^44^. In this model, direct paths from the instrument (i.e., a dichotomous variable with encouragement (Conditions B, C, D) coded as 1 and non-encouragement (Condition A) coded as 0) to the treatment variable (i.e., the number of minutes in which the participant was physically active within the 5-min window after the message) and from the treatment variable to the different outcome variables (i.e., valence, energetic arousal, calmness, and working memory, respectively) (see also Fig. 1). Random slopes were estimated for both effects, allowing for interindividual differences in the adherence effect (encouragement to treatment) and the treatment effect (treatment to outcome). Separate models were estimated for the four outcomes. In each model, a covariance was added between the residual terms of the treatment and the outcome to account for potential confounding influences on these variables. On Level 2, the two random intercepts (behavior, outcome) and the two random slopes (adherence effect; treatment effect) were allowed to covary. In addition, the Level-2 variables age (yrs), sex (male vs. female), BMI (kg/m^2^), and VO_2max_ (ml/min/kg) were added and correlations of these four covariates with the random intercepts and random slopes were estimated.

The models to test Hypothesis 2 were set up similarly. Here the dichotomous instrument was replaced by three contrasts which compared: (i) Condition A to the mean of the other three conditions; (ii) condition B (standing) to the mean of Conditions C and D (walking); and (iii) Condition C to D (walking slowly vs. fast). The treatment variable in this model was movement intensity (i.e., movement acceleration intensity in the 5 min following the prompt). Direct effects from the three contrasts to the behavior were estimated. Random slopes were estimated for the three effects as well as the effect of the treatment on the outcome^57^. In robustness analyses, we included lag-1 autoregression effects of the outcome in our models.

The convergence of the model was inspected by focusing on the potential scale reduction factor and visual inspection of trace plots, autocorrelation plots, and posterior parameter distributions. All models (Hypotheses 1 and 2) were estimated using the Bayesian estimator in Mplus Version 8.4. We used Mplus defaults regarding the number of Markov Chains and Monte Carlo chains (2), burn-in (the first 50% of estimates per chain were discarded), and weakly informative priors. In each model, we used 5000 iterations per change and a thinning factor of five. We deemed parameters to be statistically significantly different from zero when their 95% credible interval (CI) did not contain zero. Mplus input and output for all models can be accessed at https://osf.io/7vrky/. Within-subject reliability analyses were conducted in Rstudio (2023.06.0) and exploratory analyses were conducted using SPSS (v29, IBM).

## Results

### Descriptive statistics

In total, the 211 participants were prompted 19,135 times (i.e., 90.7 (SD = 21.5) prompts on average; range: 26–173). Of all prompts, 4875 (25.5%) were missed or ignored and in 14,249 situations the participants responded to the encouragement or non-encouragement message. In particular, 7177 (50.3%) of all responded prompts were randomized as a control treatment, 2366 (16.6%) as standing breaks, 2350 (16.5%) as slow walking breaks, and 2366 (16.6%) as fast walking breaks, respectively; and thus, indicating an optimal base rate of 50% for providing encouragement messages. In 42.5% of all occasions, prompts were sent between 8 a.m. and 12 pm, while 41.9% were sent in the afternoon (1 pm and 5 pm) and 15.3% in the evening (after 5 pm). Participants reported that in 54.1% of all occasions, they were at work, 40.4% at home, and 5.5% on the way, respectively.

Across all situations, the adherence rate was 81.9% (SD = 9.8%: range: 46.3–98.7%) per participant. On average, our data revealed a difference of 5.2% between non-encouragement and encouragement prompts, with a higher adherence rate for performing sedentary breaks compared to the adherence rate during control treatment. The calculated adherence rate is in line with the self-reported e-diary answers after the sedentary break treatment. In particular, participants reported in 95.5% of all standing encouragements that they performed a standing break, in 88.6% of situations a slow walking break, and in 86.3% of situations a fast walking break, respectively. In 884 cases, participants responded to the encouragement or non-encouragement message but missed or ignored the momentary e-diary ratings.

Participants reported average affective state dimension scores of 65.47 (SD = 18.33 [valence]), 56.32 (SD = 20.2 [energetic arousal]), and 60.4 (SD = 20 [calmness]); for details see Table 1). Participants completed the working memory task with an average accuracy of 89.14%. (SD = 11.82) The ICCs revealed that 60% (valence), 69% (energetic arousal), 63% (calmness), and 55% (working memory) of the variance in the e-diary ratings and tasks were due to within-subject fluctuations.

The intensity values in the 5 min after the encouragement messages indicated an accurate adherence to the study protocol with an average of 49.89 (SD = 59.0) millig during standing breaks, 214.6 (SD = 148.7) millig during slow walking breaks, and 325.1 (SD = 226.9) millig during fast walking breaks, respectively (see Table 2). E-diary questions about the performed sedentary breaks indicated that 79.9% of all breaks were taken indoors, 13.5% outdoors, and 6.6% both indoors and outdoors. Participants reported in 61.1% of all breaks that the duration was exactly 3 min, in 27.3% of all breaks longer than 3 min, and in 11.6% shorter than 3 min. Furthermore, if participants did not perform a sedentary break they stated in 45.4% of all cases that they were in a meeting. In 14.1% of all cases, the participants were not motivated to follow the encouragement message.

**Table 2:** Treatment characteristics

Treatment:	A: Sitting (Mean ± SD^a^	B: Standing (Mean ± SD)	C: Slow walking (Mean ± SD)	D: Fast walking (Mean ± SD)
Adherence [0–100%]	79.3 ± 10.5	89.4 ± 15.2	82.4 ± 20.2	81.5 ± 21.0
Valence [0–100]^b^	65.1 ± 18.6	66.0 ± 17.9	65.7 ± 17.9	66.0 ± 18.4
Energetic Arousal [0–100]^b^	55.3 ± 20.3	55.9 ± 19.6	57.0 ± 20.1	59.4 ± 20.2
Calmness [0–100]^b^	60.4 ± 20.3	61.0 ± 19.9	60.9 ± 19.2	59.0 ± 20.1
Working Memory [0–100%]^b^	89.1 ± 12.0	89.1 ± 12.2	89.3 ± 11.2	89.4 ± 11.4
Sitting/reclining/lying time [min]^c^	4.4 ± 1.3	1.8 ± 1.6	2.0 ± 1.7	1.9 ± 1.7
Steps [number]^c^	3.5 ± 11.0	4.3 ± 10.2	39.0 ± 28.6	53.7 ± 35.5
Movement intensity [millig]^c^	35.2 ± 64.9	49.9 ± 59.0	214.6 ± 148.7	325.1 ± 226.9

^a^Standard deviation,

^b^Assessed via e-diary,

^c^Moving average over the 5 min after the encouragement or non-encouragement message.

### Hypotheses 1: Causal effects of sedentary breaks on affective and cognitive parameters

The convergence of the model based on the potential scale reduction factor ranged between 1.001 and 1.018. The visual inspection of the trace plots, autocorrelation plots, and posterior parameter distributions revealed no irregularities.

As an essential precondition to test causal effects, we first focused on the effect of the encouragement or non-encouragement message on the following behavior (i.e., 5 min of being sedentary or physically active after the prompt). Across all models, the results showed that after a non-encouragement message, the participants spent 4.43 min in a sedentary position, while on average an encouragement message significantly reduced the sedentary time by *b* = 2.53 (95% CI: [2.63, 2.42]) min. The explained variance of the effects amounted to 42.8%. Hence, participants adhered to the experimental condition sufficiently well, that is, a substantial amount of the variability in the treatment behavior was experimentally induced, which allows us to estimate the causal effect of this behavior on the four outcome measures with our instrumental variable approach.

#### Valence (Hypothesis 1a)

As hypothesized, sedentary breaks positively predicted the affective dimension valence (unstandardized *b* = −0.34, 95% CI [0.17; 0.56]); that is, sedentary breaks improved upcoming valence scores. Translated to practice, breaking up sedentary behavior by 3 min on average increased valence by 1.02 points on a scale from 0 to 100. The explained variance of the effect amounted to 0.6%, suggesting a small effect of breaking up sedentary behavior on valence.

#### Energetic arousal (Hypothesis 1b)

In line with hypothesis 1b, sedentary breaks positively predicted energetic arousal (unstandardized *b* = −0.79, 95% CI [0.54; 1.04]); that is, sedentary breaks improved subsequent energetic arousal. Translated to practice, breaking up sedentary behavior by 3 min on average increased energetic arousal by 2.4 points on a scale from 0 to 100. The explained variance of the effect amounted to 1.5%, which suggests a small effect.

#### Calmness (Hypothesis 1c)

In contrast to our expectation, the model showed no significant effect of sedentary breaks on the affective dimension of calmness (unstandardized *b* = −0.09, 95% CI [−0.15; 0.31]). The explained variance amounted to 0.3%.

#### Working memory (Hypothesis 1d)

Sedentary breaks after the encouragement message did not significantly predict working memory (unstandardized *b* = −0.09, 95% CI [−0.07; 0.24]). Thus, Hypothesis 1 d was not supported. The explained variance amounted to 0.7%.

Across all models, none of the Level-2 covariates age, BMI, sex, and VO_2max_ correlated significantly with the effect of sedentary breaks on the four outcomes.

### Hypotheses 2: causal effects of sedentary break intensities on affective and cognitive parameters

The convergence of the model based on the potential scale reduction factor ranged between 1.001 and 1.002. The visual inspection of the trace plots, autocorrelation plots, and posterior parameter distributions revealed no irregularities.

As an essential precondition to test causal effects, we first focused on the effect of the different encouragement messages on the following movement intensity behavior (i.e., movement acceleration intensity in the 5 min after the prompt). The three contrasts explained 50% of the variance in the behavior. Hence, participants adhered to the different experimental conditions, and a substantial amount of the variability in the treatment behavior (i.e., movement acceleration intensity) was experimentally induced which allows us to estimate the causal effect of this behavior on the four outcome measures.

In particular, movement acceleration intensity positively predicted the affective state dimensions valence (unstandardized *b* = 0.004, 95% CI [0.001; 0.007]), and energetic arousal (unstandardized *b* = 0.013, 95% CI [0.009; 0.017]). That is, higher movement intensity increases upcoming valence and energetic arousal. Translated to practice, increasing intensity from a static posture such as standing to a dynamic movement such as walking ~400 millig increased valence on average by 1.6 points and increased arousal by 5.2 points on a scale from 0 to 100. The explained variance of the effect amounted to 3.1% (valence) and 4.6% (arousal), respectively. In contrast, movement acceleration intensity did not predict the affective state dimension calmness (unstandardized *b* = −0.002, 95% CI [−0.006; 0.002]), and performance in the working memory task (unstandardized *b* = 0.001, 95% CI [−0.002; 0.004]). Across all models, none of the Level-2 covariates age, BMI, sex, and VO_2max_ correlated significantly with the effect of movement intensity on the outcomes.

To further inspect the effects of the encouragements on the four outcomes, we conducted a series of two-level random-intercept random-slope models. First, the type of encouragement message significantly predicted the outcomes of valence and energetic arousal. In particular, encouragement messages for a sedentary break enhanced valence compared to a non-encouragement message. After Bonferroni adjustment for multiple comparisons, the mean difference of the estimated marginal means between standing and the control condition remained significant (0.97; *p* = 0.03). The model for the outcome energetic arousal revealed that encouragement messages for a sedentary break enhanced energetic arousal compared to a non-encouragement message (mean difference of the estimated marginal means to the control condition: slow walking (1.94; *p* ≤ 0.001); fast walking (4.2; *p* ≤ 0.001)). Moreover, fast walking encouragement significantly enhanced energetic arousal compared to the standing (3.24; *p* ≤ 0.001) and the slow walking (2.27; *p* ≤ 0.001) encouragement. The calmness model showed that fast walking (1.42; *p* = 0.002) decreased calmness compared to the standing (−2.21; *p* ≤ 0.001) and the slow walking (−2.27; *p* ≤ 0.001) encouragement. The analyses revealed no significant mean differences between encouragement and non-encouragement messages in the working memory outcome (for details: see Supplementary Table 1a–d). Our robustness analyses indicated that including autoregression effects of the outcome in our models, changed only the effect of movement acceleration intensity on valence from significant to non-significant, whereas all other models remained unchanged.

### Heterogeneity between participants: exploration of the sedentary breaks effect

**Fig. 2:**
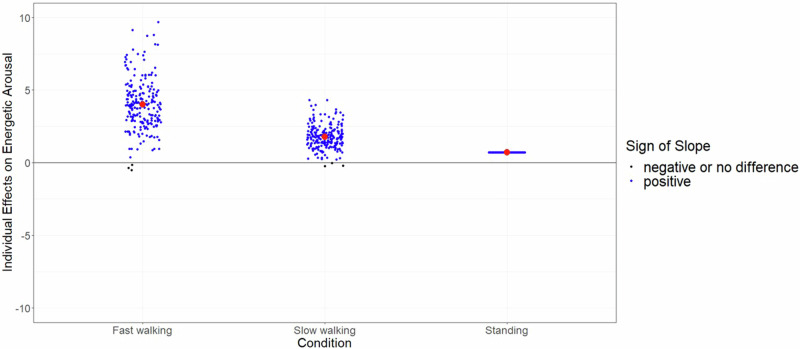
Individual effects on energetic arousal based on random slope coefficients across the different break intensities. Each dot represents the random slope coefficient for each participant. Blue dots indicate a positive effect, whereas a black dot indicates a negative or no effect. The red dot represents the average value of random slope coefficients (i.e., the fixed effect of the model).

To explore the heterogeneity between participants, we focused on the random slope coefficients derived from the multilevel model for each outcome and the three sedentary break conditions, respectively (see Fig. 2 as well as Supplementary Fig. 1a–c). The main aim of this exploratory part is to understand how different participants react to the different types of sedentary breaks. To exemplify the heterogeneity, we describe the difference in the outcome of energetic arousal in the main text. The description of the results for the outcomes valence, calmness, and working memory performance are presented in the Supplementary file. As a summary of the explorative analysis, we identified that there is an apparent variation between participants in the effect of slow and fast walking breaks on the outcome of energetic arousal.

Further, we focused on the questions of which type of break might be most valuable to the participants. None of the participants had a negative random slope coefficient after a standing break, whereas three participants had a negative sign after both slow and fast walking breaks. The positive random slope coefficients after a standing break were on average 0.709 with a range from 0.707 to 0.711, indicating a homogenous effect. The average after a slow walking break was 1.781 with a range from −0.243 to 4.318, while the average after a fast walking break was 4.000 with a range from −0.516 to 9.701 (see Fig. 2).

In a final exploratory analysis, we tested whether the self-reported well-being and sedentary behavior levels had changed at baseline and after 15 days of measurement. Independent t-tests revealed a significant increase in well-being (*t* (205) = −3.91, p < 0.001) with a small to medium effect size (Cohen’s *d* = −0.27), but no differences for the level of sedentary behavior (see Supplementary Table 2 for more details).

## Discussion

This is the first empirical study that integrated the within-person encouragement approach to test the causal effects of short 3-min sedentary breaks on affective and cognitive parameters in daily life. The setup of the study can be seen as a blueprint that the empirical application of the within-person encouragement design is feasible with excellent values in the areas of providing encouragement messages and adherence. As theoretically hypothesized, results showed that compared to uninterrupted sedentary bouts (≥30 min), short 3-min sedentary breaks had a positive causal effect on the affective state dimensions valence and energetic arousal. The explained variance across the significant findings ranged between 0.6 and 1.5%, suggesting a small effect of breaking up sedentary behavior on affective parameters. Although the association was small, the fact that it was observed over a 5-min window and will be repeatedly experienced by individuals over time in daily life suggests it could have a meaningful impact on how people feel in response to sedentary breaks and how they engage with it in the long term. Contrary to expectations, no significant effect was found for working memory performance and the affective state dimension calmness. Furthermore, as expected the variation in encouragements regarding the intensity level (i.e., standing, slow walking, and fast walking) positively predicted the following intensity behavior, and the intensity rating was linearly associated with valence and energetic arousal. Finally, the conducted explorative analyses revealed heterogeneity regarding the effects of sedentary breaks on energetic arousal and indicated high variation from person to person. In particular, the effects of the fast walking condition and the slow walking condition varied substantially in size across individuals, which leads us to assume that higher intensities could not only enable greater effects, but also larger differences between individuals.

Our study provides valuable methodological insight into approaching questions of causality in everyday life. We achieved an optimal base rate between encouragement messages (49.7%) and non-encouragement messages (50.3%), highlighting the benefits of the sophisticated approach to incorporate triggered e-diaries via continuous monitoring of sedentary behavior into the within-person encouragement design. Furthermore, the adherence rate was 81.9% across all encouragement and non-encouragement messages. This shows that while a perfect adherence rate is not realistic under daily life conditions, it is possible to achieve very high rates of adherence and thus optimal preconditions to receive adequate statistical power to detect causal effects and to estimate these effects without noticeable bias^44^. Therefore, we achieved our methodological goal, namely to provide a showcase to approach causal effects in daily life under realistic conditions of adherence. Moreover, the set-up of the study can also be seen as a template for different research questions in the area of behavioral research, focusing on causal effects in everyday life. Notably, our study was conceptualized to test the causal short-term effects of interrupting sedentary behavior, rather than to change behavior. Therefore, future research endeavors may be interested in integrating behavior change techniques. Therefore, future research endeavors may be interested in integrating behavior change techniques^58^ such as information about health consequences, goal setting, or social support into a wearable triggered e-diary system embedded in a within-person encouragement design.

The findings contribute to our understanding of the complex relationship between short sedentary breaks on both affective and cognitive parameters in daily life. This study stands out as the first empirical investigation to integrate a within-person encouragement approach, shedding light on the causal effects of brief 3-min sedentary breaks. Consistent with our hypotheses, the results revealed that compared to prolonged uninterrupted sedentary bouts of 30 min, the encouragement to perform short 3-min sedentary breaks may have elicited positive causal effects on the affective state dimensions valence and energetic arousal. These findings are partly in line with observational findings^46,47^, experimental findings in occupational settings^59–61^, and randomized trials using a between-subject experimental design^48,62^. For example, Edwardson et al.^48^ evaluated the impact of a multicomponent intervention (Stand More AT (SMArT) Work) designed to reduce sitting time and reported a significant decrease in occupational fatigue. Faulkner et al.^47^ examined the effects of a low-cost standing desk intervention to reduce occupational sitting and observed significant differences between control and intervention groups in fatigue levels, but found no effects on valence. Kuo et al.^62^ focused on the effects of a 2-min walking break compared to free-living conditions and revealed that breaking up sitting elicits greater self-perceived energy levels. Horiuchi et al.^61^ have shown that intermittent exercise breaks such as 1 min half-squats every 20 min compared to 3 h of prolonged sitting time increased mental energy levels. Our observed improvements of 1.02 points in valence and 2.4 points in energetic arousal on a 100-point scale, though modest, are comparable to other sedentary break interventions. Mailey et al.^63^ reported similar mood improvements with 3-min walking breaks, while Bergouignan et al.^59^ found significant improvements in vigor using 5-min treadmill walking breaks. Despite different scales, these studies consistently highlight the positive impact of short sedentary breaks on affect. Moreover, the brevity of our intervention (3-min breaks) makes it highly feasible to implement in various settings, from office environments to home-based work, without significantly disrupting daily routines. Our results suggest that even brief interruptions in sedentary behavior can lead to immediate changes in valence and energy levels, highlighting the potential benefits of incorporating such breaks into daily routines. Moreover, exploratory analyses suspect that these changes in energy levels reveal heterogeneity across break intensities and they were not consistently positive for all participants, which is in line with findings reported by Nagy et al.^20^. Based on an experimental approach to examine the acute effects of intermittent short sedentary breaks on mood, they found that children “felt better” after playing games on a tablet compared with being active^20^. This underscores the need for a more individualized approach. Future research could explore individual factors like participants’ perceived lack of control or sense of obligation to follow the study protocol, as well as contextual factors such as whether they are at work or at home.

Future research endeavors may be eager to understand the physiological mechanisms behind the effects of sedentary breaks on affective states in daily life. As a starting point, Chandrasekaran et al.^64^ mapped the myriad of potential physiological mechanisms of sedentary breaks on metabolic, vascular, and endocrine functions. For instance, elevated levels of brain neurotrophic factors such as dopamine and expedited anti-inflammatory functions are among the proposed mechanisms supporting enhanced cognitive performance^64^. Remarkably, some physiological responses in the context of sedentary behavior and mental health were already detected through experimental studies. Endrighi et al.^65^ have shown that increased free-living sedentary time over two weeks led to mood disturbances, irrespective of changes in physical activity levels. In particular, alterations in mood were found to be linked with stress-induced interleukin-6 responses. Therefore, focusing on inflammatory processes might be a promising avenue to understand the physiological mechanisms between sedentary breaks and changes in affective states. However, one methodological challenge is to collect physiological parameters continuously in daily life. Schenk et al.^66^ showed that the assessment of urinary inflammatory markers is feasible in an intensive day-to-day study in healthy individuals but is still limited to provide continuous information. A first indirect approach might be to collect data via continuous glucose monitoring^67^, since inflammatory markers can affect the metabolism and have an impact on blood sugar levels^68^.

Interestingly, while the positive effects on affective dimensions were evident with small effect sizes, no significant impact was observed on working-memory performance or the affective state dimension of calmness. This divergent result from our expectations refers to the complex interplay between sedentary behavior, effect, and cognitive functioning, indicating that the effects of sedentary breaks may vary across different cognitive domains and affective states. Several reasons may explain the absence of effects on these outcomes. First, a recently published overview highlighted that mechanisms between the effects of sedentary breaks on cognitive performance have been highly inconsistent^8^. The authors and further overviews^10,31^ concluded that the extent to which sedentary breaks alter cognitive function remains unclear, but the first results indicate that sedentary breaks do not negatively affect cognitive performance. Further, Pinto et al.^8^ call for a long-term investigation to understand the effects of sedentary breaks on cognitive performance. In our study, we focused on momentary working memory performance, which represents only one core executive function^34^. Since findings demonstrate that brief cognitive assessments made in uncontrolled naturalistic settings provide measurements that are to some extent comparable in reliability to assessments made in controlled laboratory environments^27^, we expect more studies with a focus on the relationship between sedentary breaks on momentary core executive functions such as mental flexibility or inhibitory control. Second, although our test specifications are based on a 100-day practical phase from a laboratory study with adults^54^, so far only a few studies^69,70^ integrated the working memory updating task as a short mobile version in an ambulatory assessment study design among university employees. On average participants achieved a score of 89% which might indicate the presence of ceiling effects for at least some participants. The resulting low within-person variability in working memory performance might have reduced the power to detect causal within-person relations with the induced treatment behavior. In future studies, the working memory task might need to be adapted to increase task difficulty to increase within-person variability in working memory performance. Third, working memory as well as affective state dimensions are also associated with other physical behaviors such as sleep or moderate-to-vigorous physical activity^71^. Although our data provide no evidence that short 3-min sedentary breaks immediately result in working-memory performance changes, the mechanisms of a potential impact can appear on a different temporal level, such as periods of three to 4 h or on a day level. In this context, a compositional data analysis approach can be used to understand the time-use composition of physical behaviors such as sitting, standing, walking, and sleeping on affective and cognitive parameters^72^. More specifically, future research endeavors might be interested in developing compositional triggered assessments and thus to intervene “just in time” not only on the basis of sedentary behavior, but also on the basis of unfavorable person-specific compositions.

Moreover, the descriptively observed heterogeneity between participants merits further discussion. We identified across all outcomes that the variance for the walking conditions was approximately five times higher compared to the standing condition, which leads us to assume that higher intensities could enable greater effects. This finding underscores the importance of tailoring sedentary break interventions to individual preferences and capabilities, as personalized encouragement strategies may yield differential outcomes based on individual characteristics, habits, and environmental factors. A central preliminary work^46^ for the conception of the presented study focused on different break patterns (i.e., frequency [number of breaks], duration [length of breaks], intensity [metabolic equivalent], and context [home vs. work] to optimally enhance affective states. The study indicated that breaking up sedentary behavior frequently and intensively, for example, by walking instead of standing, may be most beneficial, whereas break duration was not associated with affective states. This finding is in line with studies that highlighted that short break durations such as 2 or 3 min are sufficient to improve biomarkers of cardiometabolic health^73^. However, given the heterogeneity of the reported data, we expect a variation from person to person regarding the combination of frequency, duration, intensity, and context of sedentary break patterns. A promising step towards a personalized just-in-time system might be the integration of artificial intelligence to identify optimal break patterns. In general, the adoption of artificial intelligence in physical activity research started slowly^74^, but the latest published results and concepts can be seen as a promising avenue for future research efforts. For example, Yuan et al.^75^, used a 700,000 person days unlabeled dataset from the UK Biobank and showed that human activity recognition is feasible with limited labeled data or where good sampling coverage is hard to achieve. The identification of specific activities such as household activities or different exercise activities helps sharpen the recognition of break types. A future scenario that maximizes the causal effect on a personal level via artificial intelligence may integrate real-time machine learning detection and encourage participants just in time adaptively while providing the optimal break pattern (i.e., frequency, duration, intensity, context). Here, participants need to wear a wearable over a longer time frame and simultaneously capture affective states and cognitive performance. This training and learning phase would allow a second phase to predict the optimal break moments. As a final point, such a system may adapt and evolve, adjusting the encouragements for sedentary breaks to account for natural changes in behavior, such as transitioning from student to employee status

Some limitations of our work merit further discussion. First, the conceptualization of break patterns focused on different types (standing vs. walking) and different intensities (slow walking vs. fast walking) but did not differentiate regarding break duration or other potential types such as strengthening exercises. Preliminary work^46^ has indicated that frequently and intensively breaking up sedentary behavior may be most beneficial, whereas break duration was not associated with affective states. Second, we included university employees, a population at high risk of sedentary behavior. Therefore, a limitation of this study is the lack of representativeness in the sample, which may limit the generalizability of the findings to broader populations; thus, additional investigations are warranted. Third, the mobile numerical updating task indicates ceiling effects for at least some participants. Although our test specifications are based on a 100-day practical phase from a laboratory study with adults^54^, we recommend conducting a sample-specific validation study to identify optimal test specifications or to integrate an adaptive version. Fourth, the wearable triggered algorithm focused on the postural component of the sedentary behavior definition^23^, but not on the intensity component. Future refinements of the algorithm may integrate both components. Fifth, although participants were not informed of the specific hypotheses, we cannot rule out the possibility that the observed improvements in affective states due to sedentary breaks may have been influenced by participants’ expectations that the breaks would enhance their affective state, a potential example of confirmation bias. Sixth, our study design enables the analyses of the direct effects of sedentary breaks on affective states and working memory but is restricted in accounting for the complex sequential dependencies between treatment, outcome, and covariates. Thus, future research endeavors may benefit from further development of statistical models accounting for these complexities^76^.

In conclusion, our study shed light on the effects of short 3-min sedentary breaks on both affective and cognitive parameters in daily life, utilizing a within-person encouragement approach. The findings contribute valuable insights aligned with the WHO slogan “every move counts”, underscoring the importance of promoting physical activity, such as integrating sedentary breaks into daily routines. Consistent with our hypotheses, the results indicate that these brief interruptions in sedentary behavior can positively impact certain aspects of affective states, particularly valence and energetic arousal. Moreover, the findings contribute to the growing body of literature on sedentary behavior and its implications for affective parameters in daily life. By elucidating the causal effects of short sedentary breaks, this study provides valuable insights that can inform the development of targeted strategies to promote health and well-being in sedentary populations. Future research efforts may address understanding the physiological nature of causal effects, formulating specific recommendations for different target groups, and maximizing the causal effect on a personalized level using artificial intelligence approaches. With advancements in monitoring and prompting behavior, along with ambulatory assessment of outcome variables, this approach holds the promise of pioneering a new area in behavioral research, emphasizing causal effects in everyday life.

## Supplementary information

Supplementary Information

## Data Availability

The datasets used and/or analysed during the current study are available from the corresponding author on reasonable request.

## Code availability

The underlying code for this study is available in OSFHOME and can be accessed via this link https://osf.io/7vrky/.
