# Supplementary material for: Causal effects of sedentary breaks on affective and cognitive parameters in daily life: a within-person encouragement design
Source: Npj Ment Health Res. 2024 Dec 21;3:64. doi: 10.1038/s44184-024-00113-7 (PMC11662072; doi:10.1038/s44184-024-00113-7)
Supplement: Supplementary file 1 — Supplementary Information [file 44184_2024_113_MOESM1_ESM.pdf]

Two-level random-intercept random-slope models and mean difference of the estimated marginal means between different type of triggers.

Supplementary Table 1a: Pairwise comparison – Outcome: Valence

Trigger	Trigger	Mean Difference	Std. Error	Df	Sig.[a]	95% CI – lower Bound	95% CI – upper Bound
Fast walking	Slow walking	-.081	.435	13162	1.000	-1.229	1.066
	Standing	-.173	.429	13162	1.000	-1.306	.960
	Control	.793	.353	13162	.149	-.139	1.724
Slow walking	Fast walking	.081	.435	13162	1.000	-1.066	1.229
	Standing	-.091	.428	13162	1.000	-1.220	1.037
	Control	.874	.351	13162	.077	-.052	1.800
Standing	Fast walking	.173	.429	13162	1.000	-.960	1.306
	Slow walking	.091	.428	13162	1.000	-1.037	1.220
	Control	.965	.344	13162	.030	.058	1.873
Control	Fast walking	-.793	.353	13162	.149	-1.724	.139
	Slow walking	-.874	.351	13162	.077	-1.800	.052
	Standing	-.965	.344	13162	.030	-1.873	-.058

Based on estimated marginal means
The mean difference is significant at the ,05 level.
[a] Adjustment for multiple comparisons: Bonferroni.

Supplementary Table 1b: Pairwise comparison – Outcome: Energetic arousal

Trigger	Trigger	Mean Difference	Std. Error	Df	Sig.[a]	95% CI – lower Bound	95% CI – upper Bound
Fast walking	Slow walking	2.265	.513	1179.957	<.001	.908	3.621
	Standing	3.237	.507	1127.296	<.001	1.896	4.577
	Control	4.202	.421	553.589	<.001	3.087	5.316
Slow walking	Fast walking	-2.265	.513	1179.957	<.001	-3.621	-.908
	Standing	.972	.505	1118.554	.327	-.363	2.308
	Control	1.937	.419	545.462	<.001	.829	3.046
Standing	Fast walking	-3.237	.507	1127.296	<.001	-4.577	-1.896
	Slow walking	-.972	.505	1118.554	.327	-2.308	.363
	Control	.965	.411	507.473	.115	-.123	2.053
Control	Fast walking	-4.202	.421	553.589	<.001	-5.316	-3.087
	Slow walking	-1.937	.419	545.462	<.001	-3.046	-.829
	Standing	-.965	.411	507.473	.115	-2.053	.123

Based on estimated marginal means
The mean difference is significant at the ,05 level.
[a] Adjustment for multiple comparisons: Bonferroni.

Supplementary Table 1c: Pairwise comparison – Outcome: Calmness

Trigger	Trigger	Mean Difference	Std. Error	Df	Sig.[a]	95% CI – lower Bound	95% CI – upper Bound
Fast walking	Slow walking	-2.226	.485	13161	<.001	-3.507	-.946
	Standing	-2.207	.479	13161	<.001	-3.471	-.942
	Control	-1.420	.394	13161	.002	-2.460	-.380
Slow walking	Fast walking	2.226	.485	13161	<.001	.946	3.507
	Standing	.020	.477	13161	1.000	-1.240	1.279
	Control	.806	.392	13161	.237	-.227	1.840
Standing	Fast walking	2.207	.479	13161	<.001	.942	3.471
	Slow walking	-.020	.477	13161	1.000	-1.279	1.240
	Control	.787	.384	13161	.243	-.226	1.799
Control	Fast walking	1.420	.394	13161	.002	.380	2.460
	Slow walking	-.806	.392	13161	.237	-1.840	.227
	Standing	-.787	.384	13161	.243	-1.799	.226

Based on estimated marginal means
The mean difference is significant at the ,05 level.
[a] Adjustment for multiple comparisons: Bonferroni.

Supplementary Table 1d: Pairwise comparison – Outcome: Working memory performance

Trigger	Trigger	Mean Difference	Std. Error	Df	Sig.[a]	95% CI – lower Bound	95% CI – upper Bound
Fast walking	Slow walking	.072	.316	10765	1.000	-.762	.906
	Standing	.123	.312	10765	1.000	-.700	.946
	Control	.324	.257	10765	1.000	-.353	1.002
Slow walking	Fast walking	-.072	.316	10765	1.000	-.906	.762
	Standing	.051	.310	10765	1.000	-.768	.870
	Control	.253	.255	10765	1.000	-.421	.926
Standing	Fast walking	-.123	.312	10765	1.000	-.946	.700
	Slow walking	-.051	.310	10765	1.000	-.870	.768
	Control	.202	.250	10765	1.000	-.458	.861
Control	Fast walking	-.324	.257	10765	1.000	-1.002	.353
	Slow walking	-.253	.255	10765	1.000	-.926	.421
	Standing	-.202	.250	10765	1.000	-.861	.458

Based on estimated marginal means
The mean difference is significant at the ,05 level.
[a] Adjustment for multiple comparisons: Bonferroni.

Heterogeneity between participants: Exploration of the sedentary breaks effect.

Among all participants, the random intercept varies between 65.1 and 97.5. None of the participants had a negative random slope coefficient after a standing, slow, or fast walking break. The positive random slope coefficients after a standing break were on average 0.994 with a range from 0.994 to 0.994. The average after a slow walking break was 0.928 with a range from 0.928 to 0.928, while the average after a fast walking break was 0.805 with a range from 0.805 to 0.805. Indicating a homogenous effect.

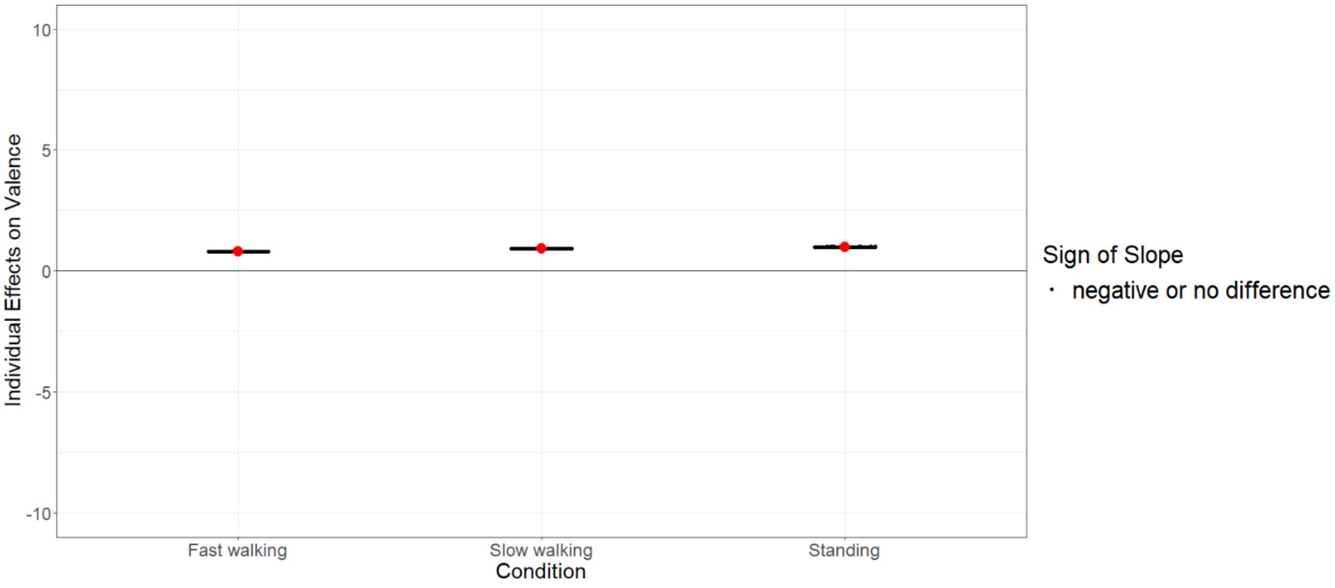

Supplementary Figure 1a: Individual effects on valence based on random slope coefficients across the different break intensities. Each dot represents the random slope coefficient for each participant. Blue dots indicate a positive effect, whereas a black dot indicates a negative or no effect. The red dot represents the average value of random slope coefficients (i.e., the fixed effect of the model).

Among all participants, the random intercept varies between 33.2 and 95.6. None of the participants had a negative random slope coefficient after a standing or slow, wheras all participants had a negative random slope coefficient after a fast walking break. The positive random slope coefficients after a standing break were on average 0.896 with a range from 0.896 to 0.869, while the average after a slow walking break was 0.920 with a range from 0.920 to

0.921. The negative random slope coefficients after a fast walking break was on average -1.329 with a range from -1.329 to -1.329. Indicating a homogenous effect.

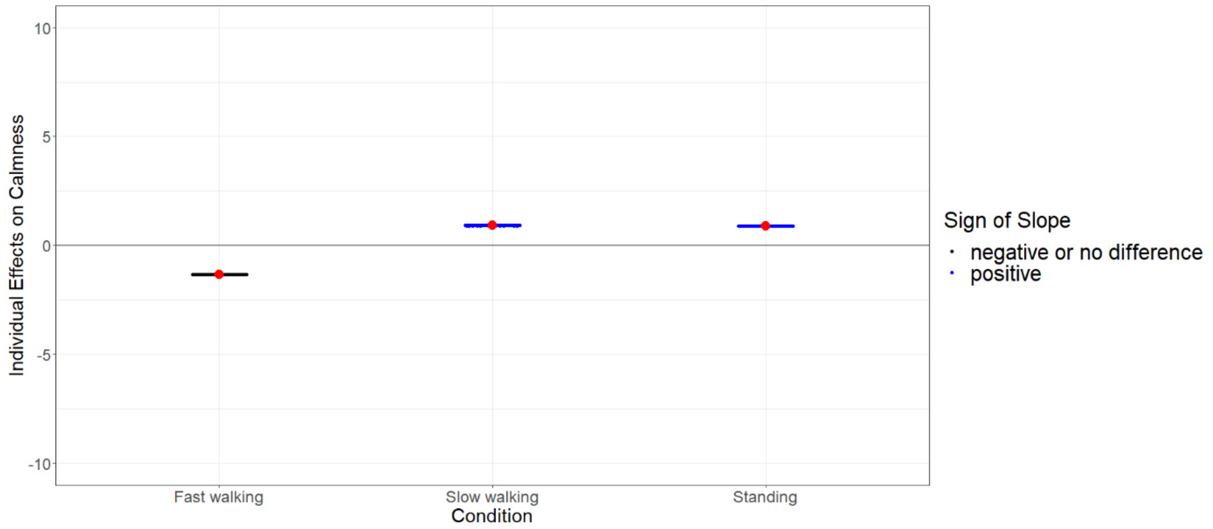

Supplementary Figure 1b: Individual effects on calmness based on random slope coefficients across the different break intensities. Each dot represents the random slope coefficient for each participant. Blue dots indicate a positive effect, whereas a black dot indicates a negative or no effect. The red dot represents the average value of random slope coefficients (i.e., the fixed effect of the model).

For working memory performance, none of the participants had a negative random slope coefficient after a standing, slow, or fast walking break. The positive random slope coefficients after a standing break were on average 0.167 with a range from 0.165 to 0.168. The average after a slow walking break was 0.255 with a range from 0.255 to 0.256, while the average after a fast walking break was 0.321 with a range from 0.321 to 0.321. Indicating a homogenous effect.

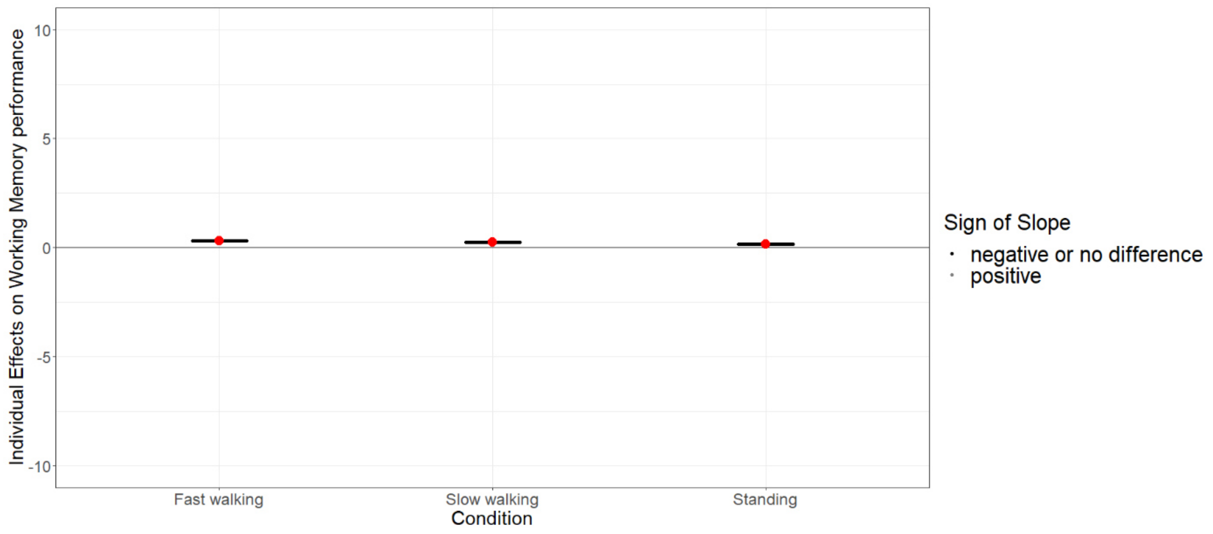

Supplementary Figure 1c: Individual effects on working memory performance based on random slope coefficients across the different break intensities. Each dot represents the random slope coefficient for each participant. Blue dots indicate a positive effect, whereas a black dot indicates a negative or no effect. The red dot represents the average value of random slope coefficients (i.e., the fixed effect of the model).

Supplement file 3 – Additional analyses

We collected self-reported questionnaire data before as well as after the ambulatory assessment phase, we now conducted exploratory analyses to test the levels of well-being and sedentary behavior via pre-post analyses. In particular, we captured well-being via the World Health Organization Well-Being Index (WHO-5). The raw score ranging from 0 to 25 is multiplied by 4 to give the final score from 0 representing the worst imaginable well-being to 100 representing the best imaginable well-being [1]. Self-reported sedentary time was assessed via a modified version of the German Sedentary Behavior Questionnaire (mSBQ) [2]. The mSBQ assessed the amount of time spent on nine context-specific sedentary behaviors: watching television (1), playing computer/video games (2), listening to music (3), talking on the phone (4), doing paperwork or computer work (5), reading a book or magazine (6), playing a musical instrument (7), doing arts and crafts (8), and driving in a car, bus or riding the train (9).

Despite observed mean differences in the descriptive statistic regarding levels of SB and well-being, t-tests revealed a significant difference for well-being ($t (205) = -3.91$, $p < 0.001$) with a small to medium effect size (Cohen's $d = -0.27$). No mean differences were observed for the level of SB before and after the ambulatory assessment phase (see Table 3).

Supplementary Table 2: T-test analysis of well-being and sedentary time before and after the ambulatory assessment phase.

	**Assessment**	**n**	**Mean ± SD**	**t**	**df**	**p (two-sided)**	**Cohen's d**
Well-being [0-100]	Pre	206	53.16 ± 12.72	-3.91	205	<.001	-0.27
	Post	206	56.20 ± 13.45				
Total SB (min/weekday	Pre	207	619.4 ± 148.7	1.51	206	0.132	0.105
	Post	207	604.9 ± 168.9				
